# A novel decision ensemble framework: Attention-customized BiLSTM and XGBoost for speculative stock price forecasting

**DOI:** 10.1371/journal.pone.0320089

**Published:** 2025-04-16

**Authors:** Riaz Ud Din, Salman Ahmed, Saddam Hussain Khan, Abdullah Albanyan, Julian Hoxha, Bader Alkhamees

**Affiliations:** 1 Artificial Intelligence Lab, Department of Computer Systems Engineering, University of Engineering and Applied Sciences (UEAS), Swat, Pakistan; 2 Department of Computer Systems Engineering, University of Engineering and Technology (UET), Peshawar, Pakistan; 3 Faculty of Computer Science and Engineering, Ghulam Ishaq Khan Institute of Engineering Science and Technology, Topi, Swabi, Pakistan; 4 College of Computer Engineering and Sciences, Prince Sattam bin Abdulaziz University, Al-Kharj, Saudi Arabia; 5 College of Engineering and Technology, American University of the Middle East, Egaila, Kuwait; 6 Department of Information Systems, College of Computer and Information Sciences, King Saud University, Riyadh, Saudi Arabia; National Institute of Technology Rourkela, INDIA

## Abstract

Forecasting speculative stock prices is essential for effective investment risk management and requires innovative algorithms. However, the speculative nature, volatility, and complex sequential dependencies within financial markets present inherent challenges that necessitate advanced techniques. In this regard, a novel framework, ACB-XDE (Attention-Customized BiLSTM-XGB Decision Ensemble), is proposed for predicting the daily closing price of speculative stock Bitcoin-USD (BTC-USD). The proposed ACB-XDE framework integrates the learning capabilities of a customized Bi-directional Long Short-Term Memory (BiLSTM) model with a novel attention mechanism and the XGBoost algorithm. The customized BiLSTM leverages its learning capabilities to capture complex sequential dependencies and speculative market trends. Meanwhile, the new attention mechanism dynamically assigns weights to influential features based on volatility patterns, thereby enhancing interpretability and optimizing effective cost measures and volatility forecasting. Moreover, XGBoost handles nonlinear relationships and contributes to the proposed ACB-XDE framework’s robustness. Furthermore, the error reciprocal method improves predictions by iteratively adjusting model weights based on the difference between theoretical expectations and actual errors in the individual attention-customized BiLSTM and XGBoost models. Finally, the predictions from both the XGBoost and attention-customized BiLSTM models are concatenated to create a varied prediction space, which is then fed into the ensemble regression framework to improve the generalization capabilities of the proposed ACB-XDE framework. Empirical validation of the proposed ACB-XDE framework involves its application to the volatile Bitcoin market, utilizing a dataset sourced from Yahoo Finance (Bitcoin-USD, 10/01/2014 to 01/08/2023). The proposed ACB-XDE framework outperforms state-of-the-art models with a MAPE of 0.37%, MAE of 84.40, and RMSE of 106.14. This represents improvements of approximately 27.45%, 53.32%, and 38.59% in MAPE, MAE, and RMSE respectively, over the best-performing attention-BiLSTM. The proposed ACB-XDE framework presents a technique for informed decision-making in dynamic financial landscapes and demonstrates effectiveness in handling the complexities of BTC-USD data.

## 1. Introduction

In today’s information-driven era, the stock market remains a global economic epicenter with far-reaching impacts on commerce, industry, and society. Traditional stock markets, characterized by established exchanges where shares of publicly traded companies are bought and sold, are integral to economic health and individual investment strategies. Predictive models have become essential tools in the financial industry to improve risk management strategies and financial decisions [1]. Speculative stocks like Bitcoin are complex and dynamic because they are inherently volatile and unpredictable. According to specific estimates (August 2024), there exist more than 13,000 (thirteen thousand) cryptocurrencies [2] with an estimated total market value of USD 2.32 trillion [3]. The share of Bitcoin is 54.8% with a value of USD 1.3 trillion. Keeping in view the market realities and trends, Bitcoin's price is highly fluctuating as per supply-demand situation, investors’ priorities, and the global economic situation [4].

Most investors depend on technical insights and market analysis to forecast and make trade-related decisions. The focus of the technical analysis is on historical price trends and data rather than market price dynamics. Indicators like moving averages are commonly used to forecast price mobility. On the other hand, fundamental perceptions were based on supply-demand factors and reliance on company reports and balance sheets. Despite their intensive use, these methods proved limited in their adaptability and compatibility to the changing nature of both conventional and predictive markets [5]. Classical time series and regression analyses emerged as viable alternative solutions to effectively address these shortfalls [6]. Different methods are used to analyze time series data and get useful statistical information, i.e., the autoregressive integrated moving average (ARIMA) model, which is used to analyze and forecast time series [7]. Though the model (ARIMA) is useful for analyzing short-term to medium-term prices, it faces challenges with the complex and non-linear pattern often prevalent in the stock markets [8].

As a result, practical and learning-oriented approaches, using machine learning (ML) like Support Vector Machine (SVM) [9] can effectively address such limitations posed by ARIMA models [10]. ML is computationally efficient enough to process and evaluate real-time stock market data. It has displayed significant effectiveness in capturing the complex nature of financial markets. Which are characterized by dynamic and multiple interactions among different elements influencing stock prices. However, ML methods are also facing difficulty with large and complex datasets [9]. Deep learning (DL), being a subset of ML, has significant improvements over conventional ML techniques [11]. The technique encompasses knowledge acquisition at different steps of description and interpretation, thus providing a more inclusive understanding of the data [9]. DL is capable of capturing complex interactions and forecasting price fluctuation in terms of historical and speculative stocks [12]. Owing to this, DL has played an important role in a variety of fields such as cancer diagnosis [13,14], detection of viral infection [15–17], cybersecurity [18,19], and intelligent transportation [20,21].

Recurrent Neural Networks (RNN) especially Long Short-Terms Memory (LSTM), associated with DL, emerge with features engineering capabilities. The models are efficient in capturing long-range dependent factors as well as temporal trends in financial time series data, thus effectively addressing memory issues [22]. Moreover, the combination of LSTM with the ML algorithm addresses correlated data extra features and improves the accuracy of stock price trends [23].

The hybrid decomposition-reconstruction model, which combines RNN with gated recurrent units (GRUs), variational modal decomposition (VMD), and sample entropy (SE), has shown significant effectiveness [24–27]. The success of hybrid models is further exemplified by the EMD-BiLSTM model, which significantly enhances forecasting accuracy. Moreover, the integration of an attention mechanism into the BiLSTM model further improves accuracy from 58.50% to 71.26% [28,29]. These developments highlight the potential of innovative model architectures and integration strategies in advancing forecasting methodologies. Moreover, hybrid methods of deep learning architectures such as CNN BiLSTM models in financial derivatives exhibit the benefits of merging the convolutional neural networks with BILSTM. CNN highlights the salient features, while BILSTM focuses on sequential dependencies. With this method, the accuracy is enhanced in various metrics [30]. The latest research demonstrates the BILSTM models’ efficacy in non-financial forecasting applications especially solar irradiation prediction as it captures both short and long-duration dependencies in a changing data scenario, resulting in more predictive accuracy [31].

In speculative stocks, the study focuses on Bitcoin price prediction, highlighting the performance of the LSTM-BTC model and its uncertainties regarding future data and generalizability to other cryptocurrencies [32]. Comparative studies using random forest regression and LSTM provide valuable insights into improving Bitcoin price prediction methodologies [33]. The importance of sample dimensions in ML algorithms for accurate Bitcoin price prediction is emphasized, with Logistic Regression and XGBoost achieving specific accuracies [34]. Bitcoin price prediction further improved with XGBoost-selected features compared to random forest (RF)--selected features [35]. Another study explores the optimization of DL models for various cryptocurrencies and particularly evaluates the performance of RNN variants such as LSTM, GRU, and BiLSTM for major cryptocurrencies [36]. While the BiLSTM has demonstrated its effectiveness in sequence modeling, it encounters difficulties when employed for stock price prediction. A significant issue lies in its uniform weight assignment to input features, neglecting the diverse levels of importance that impact stock prices.

Addressing the limitations of prior research, particularly those associated with daily trading volume and closing prices, this study proposes adopting ensemble techniques. The challenges inherent in daily trading volume and closing prices, encompassing volatility, non-linearity, external dependencies, noise, lack of clear patterns, and data quality issues, accentuate the necessity for resilient ensemble approaches to navigate the intricacies of financial market data adeptly.

The current work uses an innovative framework called ACB-XDE, which is proposed to address the Bitcoin-associated complexities in price forecasting. The framework combines an attention-customized BiLSTM with the XGBoost Algorithm to predict the daily prices of Bitcoin. It captures complex sequential dependencies and trends while the XGBoost algorithm fine-tunes predictions for improving generalization functions and performance.

The proposed ACB-XDE framework is tailored to provide a user-friendly solution for investors and financial experts through simplification of the process of Bitcoin price forecasting. It enables an individual with limited expertise to benefit from its accurate and credible prediction by harnessing advanced DL techniques. The attention system of BiLSTM constantly assigns weight to the most significant features, such as daily closing prices and daily trading volume, making the model more reliable and interpretable for users. Moreover, the iterative refinement process in XGBoost also improves prediction accuracy for providing concise, timely, and useful information.

### 1.1. Our contributions are as follows

1. The proposed ACB-XDE framework introduces an innovative approach for analyzing complex Bitcoin daily price trends by integrating an attention-customized BiLSTM with a novel attention block. This design enhances predictive performance through the refined capabilities of a modified XGBoost algorithm, offering superior trend prediction accuracy.
2. The attention-customized BiLSTM can capture complex sequential dependencies and trends in predictive market data. It also dynamically assigns weights to significant features while the new attention block improves the learning capability of the Attention-customized BiLSTM by allocating greater weightage to the daily closing of prices and daily trading volume.
3. The error reciprocal method is used iteratively to refine attention-customized BiLSTM and XGBoost prediction, thus enhancing its performance by overcoming discrepancies between theoretical expectations and actual errors.
4. Finally, the predictions are achieved through an ensemble learning approach, which integrates the individual predictions from XGBoost with attention-based customized BiLSTM models. This systematic integration enhances the diversity of predicted prices, thereby enhancing the improved generalization capabilities of the proposed ACB-XDE framework.
5. Empirical validation of the proposed ACB-XDE framework on the BTC-USD dataset exhibits improved performance, thus effectively addressing complexities and volatility reported in Bitcoin prices. Additionally, it also optimizes cost measures and shows excellent performance in recent most renowned techniques.

The forthcoming research is organized as follows: the proposed ACB-XDE framework is thoroughly explained in the Proposed ACB-XDE Framework section, which comes next. The Experimental Setup section details of the experimental procedure. The Results section presents an analysis of the proposed framework and a comparison with state-of-the-art models. Finally, the conclusion section encapsulates the study’s conclusion and potential future directions.

## 2. Proposed ACB-XDE framework

**Fig 1:**
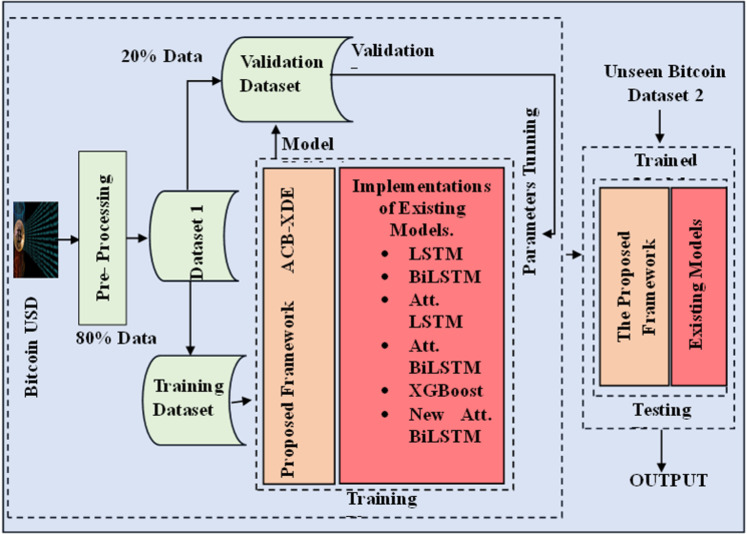
Graphical overview of the proposed study.

This paper introduces a novel DL framework, ACB-XDE, to improve the prediction of Bitcoin daily prices, which are highly volatile and complex. The proposed framework combines four distinct techniques: an attention-customized BiLSTM with an additional new attention block, an XGBoost model, a strategic error reciprocal weighting scheme, and an ensemble approach. The new attention block improves the learning capability of the attention-customized BiLSTM by giving more weight to daily closing prices and volumes. This section explains the training processes for each model, how the error reciprocal weighting is calculated, and the ensemble prediction method. Moreover, the proposed ACB-XDE framework is tested on the speculative Bitcoin dataset and compared with the best current techniques, as shown in Fig 1. Additionally, scaling is applied as preprocessing to normalize the dataset.

### 2.1. Ensemble of attention-customized BiLSTM and XGBoost

**Fig 2:**
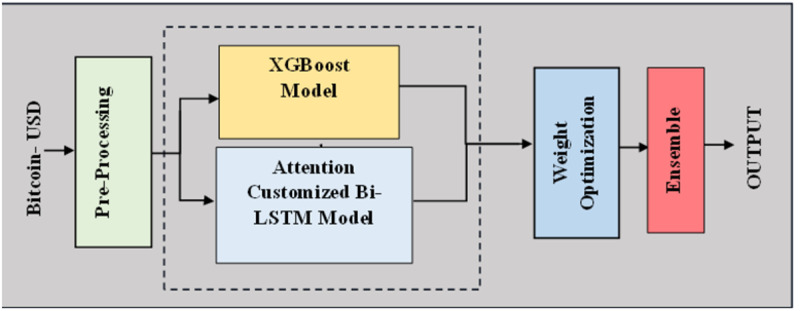
Brief Overview of the proposed Bitcoin prediction ACB-XDE framework.

**Fig 3:**
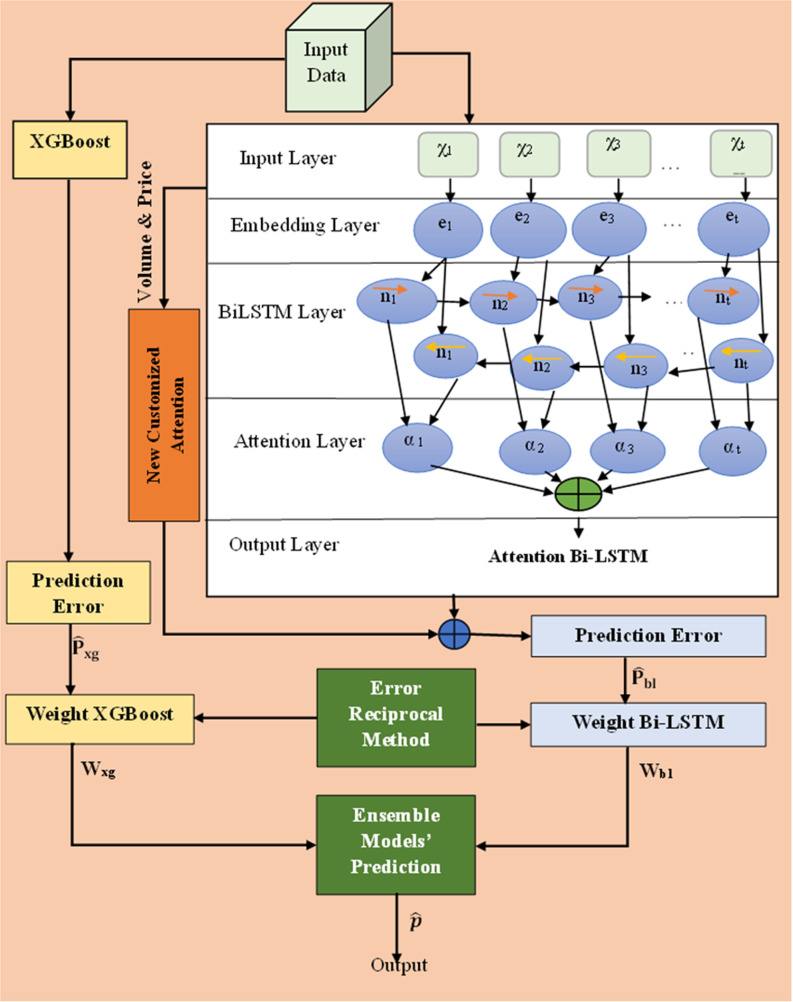
Proposed ACB-XDE detailed framework.

The ACB-XDE framework is a prediction model that combines deep learning, BiLSTM, and machine learning ensemble techniques, XGBoost. It uses a customized BiLSTM with a new attention mechanism to capture complex sequential patterns and spot market trends. This framework efficiently captures the time-based relationships in the data to improve trend detection. The addition of an XGBoost module helps improve the stability and generalizability of ACB-XDE by dealing with nonlinearities and avoiding overfitting. The error reciprocal method analyzes the predictions from the attention-customized BiLSTM and XGBoost and assigns greater weights to models with lower error rates. This weighted integration mitigates the complex limitations of individual models and offers a robust framework for tackling the multifaceted challenges of the dynamic and speculative Bitcoin market. The proposed ACB-XDE framework’s general and detailed overview is visually represented in Figs 2 and 3 respectively capitalizing on the strengths of both individual components, providing a powerful and comprehensive foundation for improved Bitcoin price forecasting.

### 2.2. Weighting method

This paper employs the error reciprocal method for weight assignment to enhance the predictive accuracy of the proposed ACB-XDE framework. The error reciprocal method plays a pivotal role in elevating prediction performance. It controls, minimizes, and optimizes the model with larger errors, which are generated by significant deviations between predicted values and actual values, by assigning inverse weight to these errors. Within the proposed ACB-XDE framework, the error reciprocal method assigns greater weights to models with smaller errors, thereby reducing the overall prediction error. The weights are calculated based on error outcomes from the primary evaluation metric, MAPE, as expressed in the following formula:


$${\hat{p}}_{\left(\text{t}\right)}{\text{=W}}_{\text{bl}}\;{\hat{P}}_{bl\left(t\right)}+{\text{W}}_{\text{xg}}\;\;{\hat{P}}_{xg\left(t\right)},\;\;\text{t=1,2,}\text{.}\;\text{.}\;\text{.}\;\text{,}\;\text{n }\;\;\;$$ (1)

$${\text{W}}_{\text{bl(t)}}=\frac{\text{E}xg}{\text{E}bl+\text{E}xg}$$ (2)

$${\text{W}}_{\text{xg(t)}}=\frac{\text{E}bl}{\text{E}bl+\text{E}xg}$$ (3)

$\hat{p}$ represents the final prediction. Wbl_(t)_ and ${\hat{P}}_{bl(t)}$ denote the weighted values and predicted values of attention-customized BiLSTM respectively. While Wxg_(t)_ and ${\hat{P}}_{xg\left(t\right)}$ represent the weighted and predicted values of XGBoost, respectively. The weight calculations are based on formulas (2) and (3), where the error values for the new attention BiLSTM and XGBoost are represented by the variables Ebl and Exg, respectively.

### 2.3. BiLSTM prediction model

The paper focuses on predicting Bitcoin (BTC) prices, proposing a novel ACB-XDE framework. The sample datasets are sourced from Yahoo Finance and encompass BTC-USD exchange rates. BiLSTM is selected because of its excellent capacity to identify patterns in sequential data and efficiently capture complex sequential dependencies, which perfectly matches the dynamic nature of the Bitcoin price [37].

In time-series financial forecasting, BiLSTM is widely used due to its effectiveness. It has also been effectively used in other non-financial domains, such as solar irradiation prediction. BiLSTM outperforms traditional approaches in the study of solar irradiation, considering both short- and long-term dependencies on structural data [31]. Additionally, integration of BiLSTM with convolutional layers and autoencoders has been shown to further enhance accuracy by extracting important hidden features from the input data and it also resolves gradient instability issues [30]. The proposed novel attention-customized BiLSTM in the ACB-XDE framework builds upon these principles by integrating novel components to enhance the model’s performance:

#### 2.3.1. Embedding layer.

The embedding layer is a fundamental component responsible for transforming raw input features such as daily closing price, daily opening price, daily trading volume, daily high, daily low, and date into a continuous vector space. The input features start as one-dimensional arrays and get converted into dense, low-dimensional vectors by the embedding layer. This modification improves the model’s ability to predict stock prices by assisting it in comprehending patterns and temporal dependencies in the data. The parameters of the embedding layer are learned along with other neural network parameters during training using backpropagation. This allows the model to adjust dynamically to the input data and pull out important features for accurate forecasting. The output from the embedding layer then goes into the next LSTM layer.

#### 2.3.2. LSTM layer.

**Fig 4:**
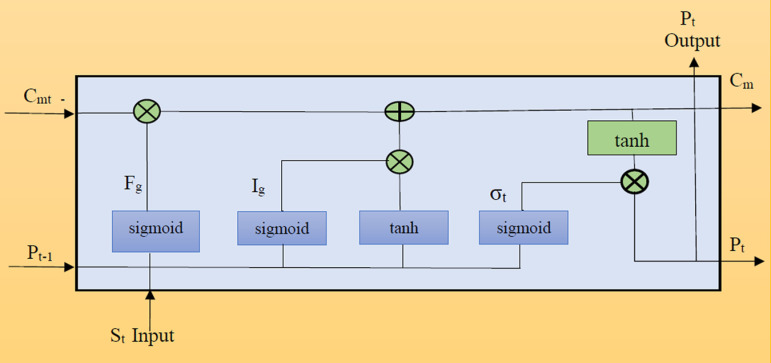
Gating mechanism of LSTM.


Recurrent neural networks (RNNs) are effective tools in the management of sequential data but their effectiveness depends more on the selection of time series range. If time series are long enough, the gradients will become smaller, thus making the learning process difficult for the framework. However, if time steps are short enough, the gradient becomes significantly large and leads to exploding gradients. Due to this phenomenon model parameters grow exponentially during backpropagation thus causing instability and preventing convergence [38]. LSTM models cater to this deficiency by solving the vanishing gradient problem through a gating system encompassing input, output, and forget gates as shown in Fig 4. These gates control the flow of information within the network and mitigate the vanishing gradient problem. The input gate controls the infusion of new information, the forget gate decides what to discard from the concealed state. While the output gate determines what information to pass on to the next layers. The LSTM layer can effectively learn long-term dependencies inside sequences due to the complex interplay between the gates. The hidden state, which connects the previous time step (H_t − 1_) with the current time step (X_t_), provides the input for the LSTM gate. Subsequently, a fully connected layer is used to calculate the LSTM’s output (6).

$${\text{Input Gate: I}}_{\text{g}}{\text{=σ(S}}_{\text{t}}{\text{WM}}_{\text{xn}}{\text{+ Ph}}_{\text{t-}}{}_{\text{1}}{\text{W}}_{\text{nIg}}{\text{+ d}}_{\text{n}}\text{)}$$ (4)

$${\text{Forget Gate: F}}_{\text{g}}{\text{=σ(S}}_{\text{t}}{\text{W}}_{\text{xf}}{\text{+ Ph}}_{\text{t-1}}{\text{W}}_{\text{nFg}}{\text{+ d}}_{\text{f}}\text{)}$$ (5)

$${\text{Gated unit: C}}_{\text{m}}{\text{=tanh(S}}_{\text{t}}{\text{WM}}_{\text{xc}}{\text{+ Ph}}_{\text{t-1}}{\text{W}}_{\text{nCm}}{\text{+ d}}_{\text{c}}\text{)}$$ (6)

$${\text{C}}_{\text{mt}}{\text{=F}}_{\text{g}}⊙{\text{C}}_{\text{m(t}}{}_{\text{-1)}}{\text{+I}}_{\text{g}}⊙{\text{C}}_{\text{m}}{}_{\text{t}}$$ (7)

$${\text{Output gate: O}}_{\text{g}}{\text{=σ(S}}_{\text{t}}{\text{W}}_{\text{og}}{\text{+ Ph}}_{\text{t-1}}{\text{W}}_{\text{nOg}}{\text{+ d}}_{\text{o}}\text{)}$$ (8)

**Fig 5:**
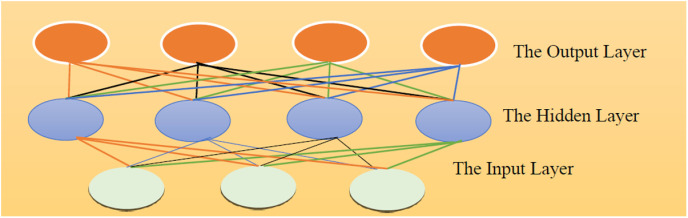
Component connection topology of LSTM.


‘Ph_t−1’_ represents the hidden state from the preceding time step, while the small set input at a given time step ‘t’ is shown by ‘S_t_’. The number of the hidden state is marked as ‘hs’. The terms ‘WM_xn_’ & ‘W_nIg_’ are the weight matrices of the input gate, while ‘σ’ is the sigmoidal function. Also, the term ‘dn’ is the offset term for the input gate. In addition, the weight matrices assigned to the forgetting gate are denoted by the symbols ‘W_xf_’ and ‘W_nFg_’ in this architecture, while the associated offset term is represented by the symbol ‘df’. The terms ‘WM_xc_’ and ‘W_nCm_’ are used as weight matrices for the gated unit, while ‘dc’ stands for the related offset term. The term ‘C_m_’ is introduced to describe the candidate _m_emory cells. Furthermore, ‘C_mt_’ designates the cell state for the current time step, while ‘C_m(t−1)_’ represents the cell state for the previous time step. Finally, ‘do’ is the offset term that corresponds to the defined weight matrices ‘W_og_’, which are linked to the output gate. The information flow in the hidden state is regulated by the multiplication of elements (x) and using the activation function (tan h) with a value range of [-1,1]. The output gate Og handles the flow of information from the memory cell to the hidden state and the final enriched output Fo is denoted (Eq. 9) and Fig 5 illustrates the components’ connection topology of BiLSTM [30].

$$\text{Fo = Og}\left(\text{x}\right)\text{tan h}\left(\text{Cm}\right)$$ (9)

The proposed ACB-XDE framework employs a specialized feature extraction method, leveraging the comprehensive information within the data to capture insights from both forward and backward perspectives. The outcomes of this two-way extraction are harmoniously combined and summarized in two dimensions. The inherent influence of the order of inputs on a single LSTM is mitigated by strategically merging the data and therefore enhancing the overall comprehensiveness of the outputs. This methodological foundation serves as a robust foundation and effectively tackles challenges related to gradient instability and sequential feature extraction in speculative stock price prediction endeavors.

#### 2.3.3. Attention mechanism of BiLSTM.

**Fig 6:**
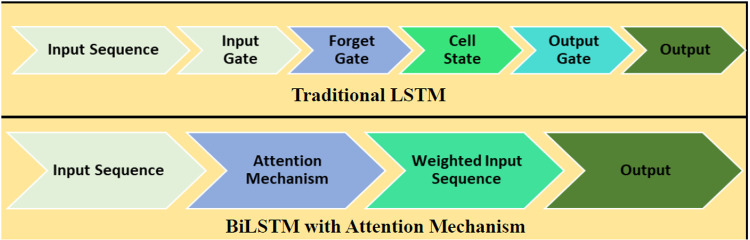
Traditional LSTM vs BiLSTM with an attention mechanism.

The new attention mechanism further refines the traditional BiLSTM to enhance the framework’s ability to capture critical market signals. The BTC-USD dataset often contains subtle features and patterns with varying degrees of importance. BiLSTM’s attention mechanism dynamically allocates weights to significant features and as a result, improves interpretability and optimizes cost measures and volatility forecasts [39]. The foundational concept behind the attention mechanism is inspired by human attention dynamics. In human information processing, attention is selectively focused on key elements rather than uniformly distributed across all information. Integrating the attention mechanism into prediction models mirrors this cognitive approach and enables the assignment of distinct weights to data. This dynamic allocation mitigates the undue influence of certain input data on the output, amplifying the significance of pivotal information. Within the customized BiLSTM model, two pivotal attention strategies emerge. Notably, the attention gate replaces the conventional forget gate seen in traditional LSTM models as illustrated in Fig 6. This gate exclusively attends to historical cell states, untied from the current input, leading to a notable reduction in overall training parameters. Additionally, the BiLSTM model employs an attention-weighting method on the model’s output. This strategic application allows for the precise identification and utilization of the most crucial and influential information [29].

### 2.4. New attention block

**Fig 7:**
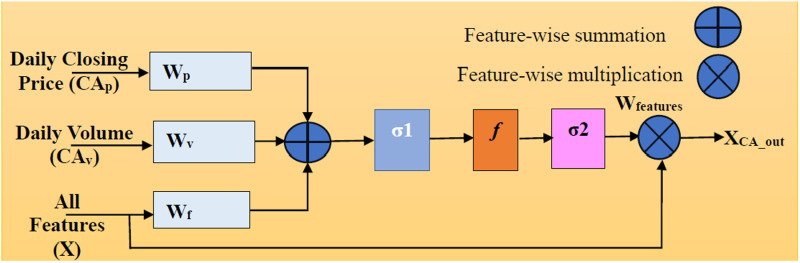
Structure of new customized-attention mechanism.


A newly employed attention mechanism enhances the learning capability of the proposed framework by allocating significant weight to daily closing price and daily trading volume. These indicators are strongly correlated with market trends and investor sentiment, and they reflect the critical aspects of market activity such as price movements and trading liquidity. The daily closing price encapsulates all intraday fluctuations, reflecting the asset’s final trading value for the day, while the daily trading volume indicates the number of shares, highlighting market interest and liquidity. Emphasizing these indicators allows the model to capture direct signals of market trends and potential price movements. Empirical studies have shown that daily closing price and trading volume are reliable predictors of future price movements. Allocating larger weight to these features leverages their predictive power, leading to more accurate forecasts. Market data includes various features that might introduce noise into the model. The new attention mechanism reduces the impact of less significant data by focusing on the most relevant features, namely daily closing price and daily trading volume. This approach enhances the model’s clarity and predictive accuracy. Moreover, the dynamic nature of the attention mechanism allows it to adjust the weight of daily closing price and daily trading volume in response to changing market conditions. This adaptability ensures that the model remains robust and accurate, even in volatile market environments. The detail of the newly employed attention mechanism block is illustrated in Fig 7. *W*_*f*_ demonstrates the all-input feature and *W*_features_ is the features-weightage coefficient at the range of [0, 1] (Equation (10)). The output *X*_*CA_out*_ highlights the price pattern while suppressing the irrelevant features. In Equations (11) and (12), σ1 and σ2 is the Relu and Sigmoid activation functions, respectively. While bC*A* and b*f* are biases, and *W*_v_, *W*_p_, and *f* are the linear transformation.

$$X_{CA\_out=}W_{features*}._{X}$$ (10)

$$X_{relu}=\sigma 1(CA_{p}W_{p}+CA_{p}W_{v}+W_{f}X+WCA)$$ (11)

$$W_{feature}=\sigma 2(f(X_{relu})+bf)$$ (12)

### 2.5. XGBoost forecasting model

In the proposed ACB-XDE framework, XGBoost is used for robust gradient-boosted decision tree implementation. XGBoost excels in handling diverse data types, managing nonlinear relationships, and preventing overfitting. XGBoost ensures model stability and generalizability thereby addressing the limitations of over-reliance on sequential information and enhancing model interpretability. Furthermore, XGBoost’s unique ability to combine weak learners facilitates the capture of complex dynamic patterns, contributing to more accurate predictions even during significant market shifts [40]. XGBoost represents a refined instantiation of the gradient-boosting decision tree paradigm, distinguished by its ability to improve predictive speed and efficiency. XGBoost employs a meticulously crafted decision tree constructed iteratively by adding trees and progressively partitioning features. This method helps the model better capture complex patterns in the Bitcoin price data [41]. It involves creating a new function, *f(x),* to represent the residual error from the previous prediction as part of the incremental integration process. Once all of the k trees in the training process are used, each tree converges to a different leaf node, and every leaf node has a unique score. The final prediction for a specific sample is achieved by summing the scores from all the contributing trees. The formal statement of the XGBoost is as follows:


$${\hat{p}}_{i}=\sum_{j=1}^{n}\omega_{j}p_{ij}$$ (13)

Herein, the forecast value is indicated by ${\hat{p}}_{i}$, sample data is indicated by $p_{ij}.$ While *n* indicates the total number of trees and $\omega_{j}$ indicates the weight.

A new tree is slowly added to the existing tree structure in each iteration cycle to model the residual disparity between the results and the predictions of the previous tree. The following Equation (14) is an explanation of the iterative process while Equations (15) and (16) state the objective function of XGBoost. $\text{Ω}\left(f_{i}\right)$ is the regularization term in Equations (15) and (16).


$${\hat{p}}_{i}^{\left(0\right)}=0$$ (14)

$$\begin{array}{l} {\hat{p}}_{i}^{\left(1\right)}=f_{1}\left(p_{i}\right)={\hat{p}}_{i}^{\left(0\right)}+f_{1}\left(p_{i}\right)\\ {\hat{p}}_{i}^{\left(2\right)}=f_{1}\left(p_{i}\right)+f_{2}\left(p_{i}\right)={\hat{p}}_{i}^{\left(1\right)}+f_{2}\left(p_{i}\right)\\ \cdots \\ {\hat{p}}_{i}^{\left(t\right)}=\sum_{k=1}^{t}f_{k}\left(p_{i}\right)={\hat{p}}_{i}^{\left(t-1\right)}+f_{t}\left(p_{i}\right) \end{array}$$

$$\begin{array}{rr} & \begin{array}{rr} & Obj\left(t\right)=\overset{n}{\underset{i=1}{\sum }}l\left(p_{i},{\hat{p}}_{i}^{\left(t\right)}\right)+\overset{t}{\underset{i=1}{\sum }}\text{Ω}\left(f_{i}\right)\\ & =\overset{n}{\underset{i=1}{\sum }}l\left(p_{i},{\hat{p}}_{i}^{\left(t-1\right)}+f_{t}\left(p_{i}\right)\right)+\text{Ω}\left(f_{t}\right) \end{array} \end{array}$$ (15)

$$\text{Ω}f\left(t\right)=\gamma L+\frac{1}{2}\lambda \overset{T}{\underset{j=1}{\sum }}\omega_{j}^{2}$$ (16)

${\hat{p}}_{i}^{\left(t\right)}$ represents the model after *t* training rounds, ${\hat{p}}_{i}^{\left(t-1\right)}$ signifies the retained function from the earlier round, whereas $f_{t}\left(p_{i}\right)$ is the recently introduced function. It is important to find *f*t that minimizes the chosen target function. Equation (15) has a regularization term called $\sum_{i=1}^{t}\text{Ω}\left(f_{i}\right)$, which affects the objective function’s tree’s complexity. Improved generalization ability and decreased complexity are correlated with a lower value of $\text{Ω}\left(f_{t}\right)$. Equation (16) shows that L is the number of leaf nodes, *ω* is the score awarded to a leaf node, γ controls the number of leaf nodes, and λ limits the scores of leaf nodes to avoid unnecessarily high values.

Second-order expansion of Taylor at *f*_*t*_ = 0 is used to determine *f*_*t*_ that minimizes the goal function. Approximation resulting from the objective function is expressed as:


$$\tau^{\left(t\right)}≃\sum_{i=1}^{n}\left[l\left(p_{i},{\hat{p}}_{i}^{\left(t-1\right)}\right)+g_{i}f_{t}\left(p_{i}\right)+\frac{1}{2}h_{i}f_{t}^{2}\left(p\right)\right]+\text{Ω}\left(f_{t}\right)$$ (17)

Here, $fd_{i}=\partial_{\hat{p}}\left(t-1\right)l\left(p_{i},{\hat{p}}^{\left(t-1\right)}\right)$, and $sd_{i}=$$\partial_{{\hat{p}}^{\left(t-1\right)}}^{2}l\left(p_{i},{\hat{p}}^{\left(t-1\right)}\right)$ is the first and second derivatives respectively. These elements are immediately eliminated because the residual error of p and the prediction scores from the original t-1 tree have no bearing on the optimization of the objective function. As a result, more simplification of the goal function produces:


$${\tilde{\tau }}^{\left(t\right)}=\sum_{i=1}^{n}\left[fd_{i}f_{t}\left(p\right)+\frac{1}{2}sd_{i}f_{t}^{2}\left(p_{i}\right)\right]+\text{Ω}\left(f_{t}\right)$$ (18)

Equation (18) aggregates the values of the loss function for every sample, hence simplifying the objective function. Then, to simplify and rephrase the goal function, samples belonging to the same leaf node are rearranged using Equation (19). The steps involved are as follows:


$$\begin{array}{rr} Obj^{\left(t\right)} & ≃\overset{n}{\underset{iz=1}{\sum }}\left[fd_{i}f_{t}\left(p_{i}\right)+\frac{1}{2}\text{sd}f_{t}^{2}\left(p_{i}\right)\right]+\text{Ω}\left(f_{t}\right)\\ & =\overset{n}{\underset{i=1}{\sum }}\left[fd_{i}l_{q_{\left(p_{i}\right)}}+\frac{1}{2}sd_{i}l_{q_{\left(p_{i}\right)}}^{2}\right]+\gamma L+\lambda \frac{1}{2}\overset{L}{\underset{j=1}{\sum }}l_{j}^{2}\\ & =\overset{L}{\underset{j=1}{\sum }}\left[\left(\underset{i\in I_{j}}{\sum }fd_{i}\right)l_{j}+\frac{1}{2}\left(\underset{i\in I_{j}}{\sum }sd_{i}+\lambda \right)l_{j}^{2}\right]+\gamma L \end{array}$$ (19)

Hence, by reformulating the previously provided formulas, the objective function transforms into a univariate quadratic function centered on the leaf node fraction ω. This transformation enables the utilization of the vertex formula to readily ascertain the optimal ω and its corresponding value for the objective function. The optimal ω j * and related objective function values for the recalibrated univariate quadratic function centered around the leaf node fraction ω can be obtained in the following manner:


$$\omega_{j}^{\text{*}}=-\frac{\sum_{i\in I_{j}}fd_{i}}{\sum_{i\in I_{j}}sd_{i}+\lambda }$$ (20)

$$Obj=-\frac{1}{2}\sum_{j=1}^{L}\frac{{\left(\sum_{i\in I_{j}}fd_{i}\right)}^{2}}{\sum_{i\in I_{j}}sd_{i}+\lambda }+\gamma L$$ (21)

$$v^{n}=\frac{v-v_{\text{min}}}{v_{\text{max}}-v_{\text{min}}}$$ (22)

For effective computations and adherence to data input specifications, data normalization is a prerequisite. Cryptocurrency data is normalized using the formula [19], effectively confining the data within the [0, 1] range. In the above formula [21], *v* and $v^{n}$, respectively denote the stock data value before and after normalization. $v_{\text{min}}$ and $v_{\text{max }}$ represent the minimum and maximum values of the stock data before normalization.

### 2.6. Models training and testing

**Fig 8:**
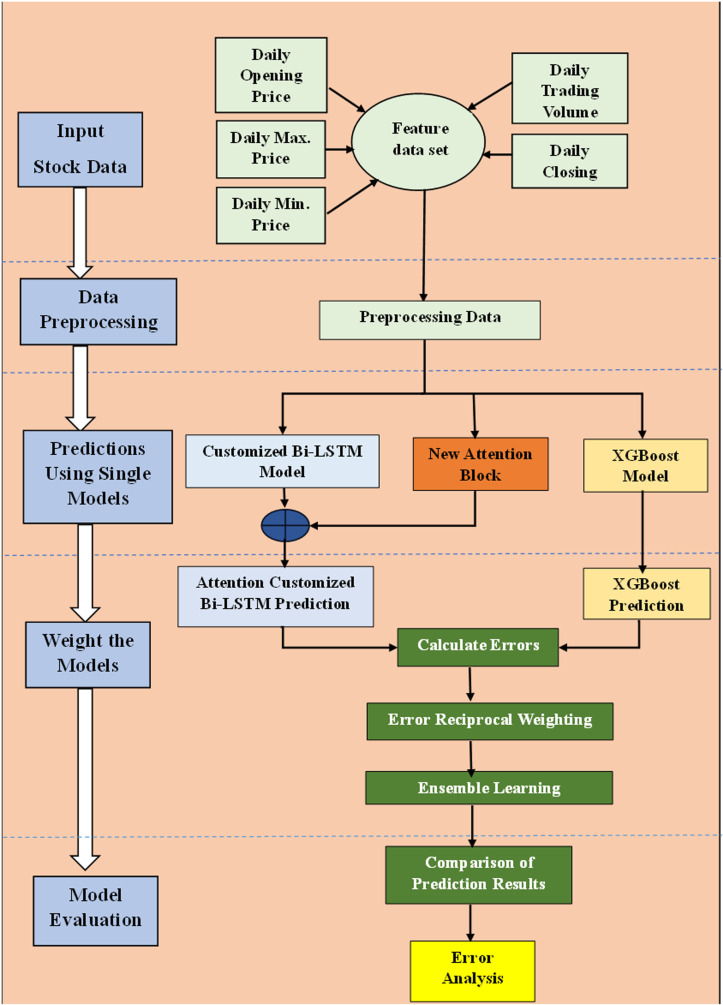
Stock price prediction methodology in the proposed ACB-XDE framework.

Initially, the attention-customized BiLSTM and XGBoost models undergo training and testing individually, and then their respective predictions are combined into an ensemble model, as illustrated in Fig 8. The prediction method consists of four distinct stages, explained as follows:

#### 2.6.1. Stage 1: Data preprocessing.

The initial stage entails comprehensive data preprocessing to enhance generalization. Selecting key features, like daily opening price, daily maximum and minimum price, daily trading volume, and daily closing price, is crucial at this stage. This meticulous selection ensures that the data is well-prepared for subsequent analysis. The culmination of the preprocessing stage involves the normalization of the input data to ensure that the input data is appropriately scaled for further analysis.

#### 2.6.2. Stage 2: Models prediction.

In the second stage, the new attention mechanism and the customized BiLSTM model are utilized to predict Bitcoin prices. The new attention mechanism focuses on daily trading volume and daily closing price, thus capturing critical market signals. This strategic emphasis can indicate unsustainable trends, such as a declining price coupled with increasing or stagnant trading volume, suggesting a transient downturn. One of the most important indicators of market sustainability is the direct relationship between daily closing price and daily trading volume. The predictions from the new attention mechanism and the customized BiLSTM model are then combined to obtain a consolidated result. This ensemble approach leverages the strengths of each method, combining the market signal detection capabilities of the attention mechanism with the sequence learning capabilities of BiLSTM. Additionally, the XGBoost model is used independently for Bitcoin price prediction. XGBoost gradient boosting framework effectively handles overfitting through regularization techniques such as L1 and L2 regularization.

#### 2.6.3. Stage 3: Assigning weights to models and ensemble models.

In stage 3, the error reciprocal approach is used to assign weights to the predictions made by the new attention-customized BiLSTM and XGBoost models based on their projected errors. This approach ensures that each model is provided with an appropriate weight and their results are ensembled to acquire the final Bitcoin price prediction. The results are compared with state-of-the-art models for assessing the performance of the proposed framework.

#### 2.6.4. Stage 4: Evaluating prediction performance.

The performance efficiency of the ACB-XDE framework is assessed through a comparison of its prediction errors with six state-of-the-art models. The improvement in Bitcoin price prediction is observed in this step.

## 3. Experimental setup

This section describes the methodology, data collection, preprocessing, model training, and hardware configuration for the experimental setup used to assess the ACB-XDE framework.

### 3.1. Evaluation methodology

Python-based simulations are employed to conduct a comprehensive evaluation of various forecasting techniques. The proposed ACB-XDE framework is compared against established state-of-the-art models, including the attention-customized BiLSTM, XGBoost, LSTM, attention-LSTM, BiLSTM, and attention-BiLSTM.

### 3.2 Data acquisition and preprocessing

#### 3.2.1. Data source.

**Fig 9:**
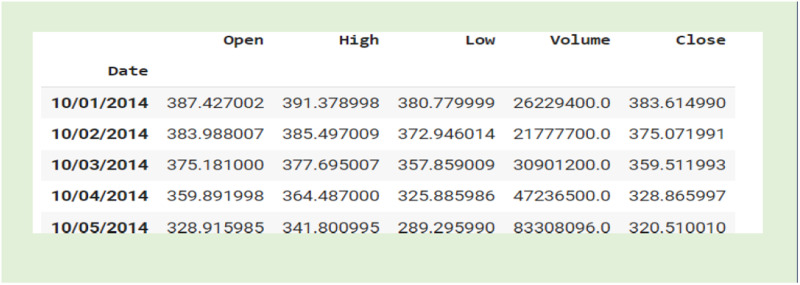
Structure of the data.

A comprehensive assessment of data from various sources shows that Yahoo Finance offers the most current and reliable dataset for evaluating the ACB-XDE framework. The chosen dataset encompassed the following features: date, daily opening price, daily maximum price, daily minimum price, daily trading volume, and daily closing price. The structure of the data is illustrated in Fig 9.

The dataset is publicly available on Yahoo Finance and the authors accessed it through the publicly accessible link given below:

https://finance.yahoo.com/quote/BTC-USD/history/?period1=1410912000&period2=1556053200&interval=1d&filter=history&frequency=1d;

To further simplify access without restrictions, we have also made the dataset available on GitHub at the following link:

https://raw.githubusercontent.com/itsriaz/PricePrediction/refs/heads/main/Dataset1.csv

https://raw.githubusercontent.com/itsriaz/PricePrediction/refs/heads/main/Dataset2.csv

#### 3.2.2. Data cleaning, handling missing values, and outlier detection.

The dataset is obtained from Yahoo Finance and it goes through extensive examination to determine its accuracy and completeness. The dataset is complete and doesn’t need any techniques such as forward filling to fill any missing values. Key summary statistics such as count, mean, standard deviation, and percentiles—minimum, 25th, 50th, 75th, and maximum values are calculated. These statistics provide insight into the data distribution before and after preprocessing as shown in Table 1.

**Table 1:** Summary of data before and after preprocessing.

Before preprocessing					
	Open	High	Low	Volume	Close
**Count**	2960	2960	2960	2960	2960
**Mean**	12843.47223	13171.35976	12477.84958	16230691935	12848.82068
**Std**	16343.51073	16762.73778	15858.59123	19925377254	16338.97055
**Min**	176.897003	211.731003	171.509995	5914570	178.102997
** 25%**	671.3450015	682.4152528	660.1447295	100269424	672.8440097
** 50%**	6912.899902	7140.667969	6752.460205	6951184896	6921.785157
** 75%**	15983.71778	16599.17529	15242.63159	28077637142	16076.05396
**Max**	67549.73438	68789.625	66382.0625	3.51E+11	67566.82813
**After preprocessing**					
	**Open**	**High**	**Low**	**Volume**	**Close**
**Count**	2960	2960	2960	2960	2960
**Mean**	0.188007151	0.188976768	0.185866741	0.046225216	0.188024297
**Std**	0.242583085	0.244433546	0.239517579	0.056768413	0.24245852
**Min**	0	0	0	0	0
** 25%**	0.007338981	0.006863498	0.007380013	0.000268822	0.007341599
** 50%**	0.099980989	0.101037471	0.09939428	0.019787428	0.100071372
** 75%**	0.234617116	0.238961032	0.227624163	0.079977765	0.235914108
**Max**	1	1	1	1	1

**Fig 10:**
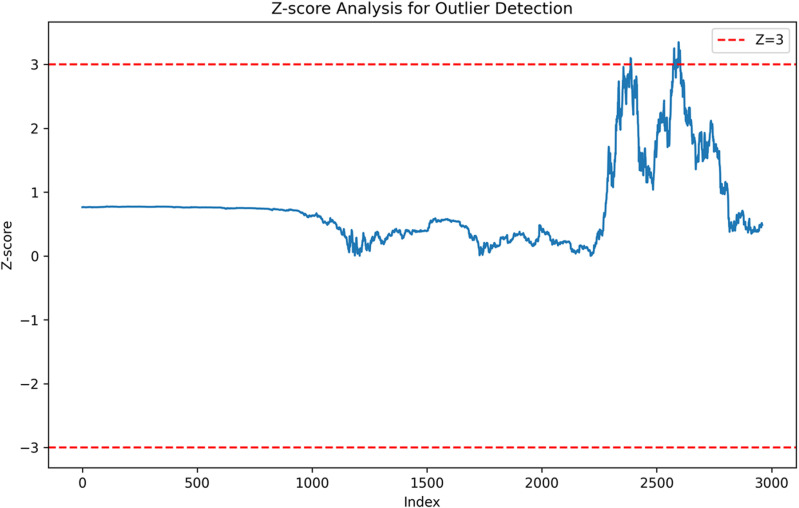
Z-score analysis for outlier detection.

Additionally, Z-score outlier detection is used to differentiate between genuine price movement and potential data errors. A total of 21 outliers are detected in the Close price based on Z-scores exceeding ± 3 as shown in Table 2 and Fig 10. However, after checking these 21 outliers are confirmed to be genuine price movements, and therefore, they are retained to preserve the dataset’s integrity.

**Table 2:** Outliers Z score greater than 3.

	Date	Open	High	Low	Volume	Close
2386	4/13/2021	59890.01953	63742.28516	59869.95703	69983454362	63503.45703
2387	4/14/2021	63523.75391	64863.09766	61554.79688	77451779687	63109.69531
2388	4/15/2021	63075.19531	63821.67188	62208.96484	60954381579	63314.01172
2574	10/18/2021	61548.80469	62614.66016	60012.75781	38055562075	62026.07813
2575	10/19/2021	62043.16406	64434.53516	61622.93359	40471196346	64261.99219
2576	10/20/2021	64284.58594	66930.39063	63610.67578	40788955582	65992.83594
2577	10/21/2021	66002.23438	66600.54688	62117.41016	45908121370	62210.17188
2581	10/25/2021	60893.92578	63729.32422	60691.80078	31064911614	63039.82422
2585	10/29/2021	60624.87109	62927.60938	60329.96484	36856881767	62227.96484
2586	10/30/2021	62239.36328	62330.14453	60918.38672	32157938616	61888.83203
2589	11/02/2021	60963.25391	64242.79297	60673.05469	37746665647	63226.40234
2590	11/03/2021	63254.33594	63516.9375	61184.23828	36124731509	62970.04688
2594	11/07/2021	61554.92188	63326.98828	61432.48828	24726754302	63326.98828
2595	11/08/2021	63344.06641	67673.74219	63344.06641	41125608330	67566.82813
2596	11/09/2021	67549.73438	68530.33594	66382.0625	42357991721	66971.82813
2597	11/10/2021	66953.33594	68789.625	63208.11328	48730828378	64995.23047
2598	11/11/2021	64978.89063	65579.01563	64180.48828	35880633236	64949.96094
2599	11/12/2021	64863.98047	65460.81641	62333.91406	36084893887	64155.94141
2600	11/13/2021	64158.12109	64915.67578	63303.73438	30474228777	64469.52734
2601	11/14/2021	64455.37109	65495.17969	63647.80859	25122092191	65466.83984
2602	11/15/2021	65521.28906	66281.57031	63548.14453	30558763548	63557.87109

### 3.3. Data splitting and preprocessing

**Fig 11:**
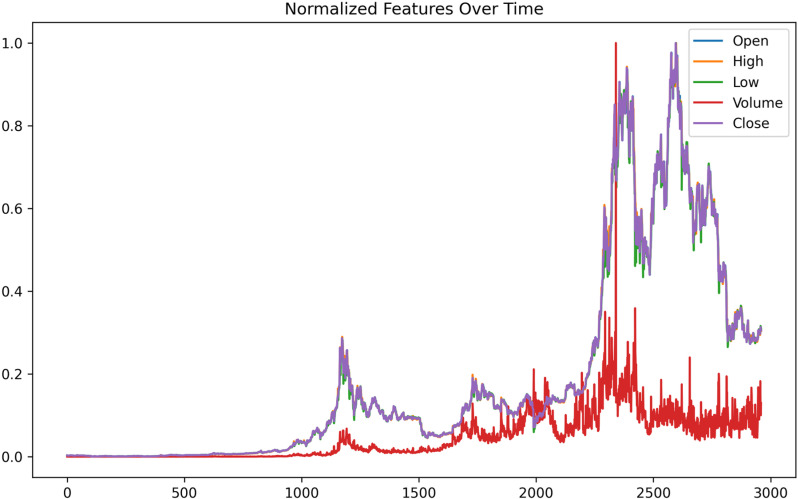
Normalized features over time.

Two distinct datasets are utilized for this research work. Dataset 1 covers the period from October 1st, 2014, to November 7th, 2022 while Dataset 2 covers the period from November 8th, 2022, to August 1st, 2023. For smooth model training and balance learning, the selected features are normalized using MinMaxScaler(), which scales values within the range of [0, 1] as shown in Fig 11, and the target variable is set as the daily close price.

### 3.4. Models training and evaluation

The attention-customized BiLSTM and XGBoost models are trained on 80% of the historical daily Bitcoin price data from Dataset 1. The remaining 20% is used for testing their performance. Subsequently, these pre-trained models are utilized for prediction on Dataset 2. The predicted values from both models are then fed into a pre-trained ensemble linear regression model to generate the final forecast. The model’s effectiveness is assessed through a comparative analysis between the final predictions and the actual values from Dataset 2. This analysis includes a thorough examination of the prediction errors.

### 3.5. Assessment criteria

The primary aim is to evaluate how well the ACB-XDE framework predicts outcomes. This evaluation uses three well-known statistical metrics. The main metric is the Mean Absolute Percentage Error (MAPE), which measures the average absolute percentage difference between predicted and actual values. MAPE is particularly useful in financial contexts as it shows prediction accuracy relative to the actual values. Additionally, auxiliary metrics such as the Root Mean Squared Error (RMSE) and Mean Absolute Error (MAE) are used in the evaluation process. RMSE highlights the spread of errors across the dataset by penalizing larger deviations more heavily than smaller ones. This metric is invaluable for identifying outliers or significant disparities between predicted and actual values. MAE offers a straightforward measure of prediction accuracy by calculating the average absolute difference between predicted and actual values. Its utility lies in providing an easily interpretable metric that considers all errors equally, irrespective of their magnitude or direction.

In Equations 23, 24, and 25, n is the amount of bitcoin data, $r_{t}$ is the real stock price, and ${\hat{p}}_{t}$ is the predicted stock price data. Using MAPE as the main evaluation metric, along with RMSE and MAE as auxiliary metrics, provides a thorough assessment of the ACB-XDE framework.


$$X_{\text{MAPE}}=\frac{1}{n}\sum_{t=1}^{n}\frac{\left|r_{t}-{\hat{p}}_{t}\right|}{r_{t}}$$ (23)

$$X_{\text{MAE}}=\frac{1}{n}\sum_{t=1}^{n}\left|r_{t}-{\hat{p}}_{t}\right|$$ (24)

$$X_{\text{RMSE}}=\sqrt{\frac{1}{n}\sum_{t=1}^{n}{\left(r_{t}-{\hat{p}}_{t}\right)}^{2}}$$ (25)

$$Confidenceinterval\left(CI\right)=MAPE\pm Z_{\alpha /2}\times \frac{\sigma MAPE}{\surd n}$$ (26)

These metrics together offer a well-rounded view of prediction accuracy, helping in robust evaluations and informed decisions on the framework’s effectiveness. Furthermore, the statistical analysis is also performed with 95% confidence intervals (CI) [42] based on the metric of MAE as the main metric. This step enables considering variability in the predictions and, therefore, highlights the stability and consistency of the performance of the model from trial to trial. Variations in MAPE for each model are calculated over the 10 independent runs/iterations to ensure the sufficiency and reliability of the assessment in terms of the predictive capabilities of the proposed ACB-XDE framework. In Equation 26, MAPE is the Mean Absolute Percentage Error, $Z_{\alpha /2}$ is the Z-score corresponding to the desired confidence level (for 95%, $Z_{\alpha /2}$ = 1.96), σMAPE is the standard deviation of the MAPE values and *n* is the number of samples (iterations or data points).

### 3.6. Hardware setup

The experimental hardware platform consists of an AMD Ryzen 7 4800H with Radeon Graphics (16 CPUs) running at 3GHz and 32GB RAM. The proposed ACB-XDE framework is implemented in Python, utilizing the py-XGBoost framework for XGBoost and the Keras DL framework for the attention-customized BiLSTM model.

### 3.7. Parametric configurations

This section delves into the configurations necessary for fine-tuning both the BiLSTM and XGBoost models. The choice of parameters significantly impacts the model’s predictive accuracy and generalization capabilities. Therefore, understanding the intricacies of model configuration is paramount for achieving optimal performance.

The choice of parameters significantly impacts the model’s predictive accuracy and generalization capabilities. Therefore, understanding the intricacies of model configuration is paramount for achieving optimal performance.

#### 3.7.1. Attention-customized BiLSTM.

The performance of the attention-customized BiLSTM model is significantly influenced by factors such as the number of units, input feature dimension, and the number of layers. Detailed parameters configuration for this model is provided in Table 3.

**Table 3:** Attention-customized BiLSTM parameter settings.

Model	Parameter	Description	Value	Explanation
XGBoost	reg	Objective	Squared error	To perform regression and minimize the mean squared error during the training process.
	n_estimators	The number of decision trees	100	This parameter holds significant influence, capable of adjusting the model to its maximum extent in a single instance.
	max_depth	The maximum depth of	8	The typical range is between 10 and 100, and adjustments can be made as needed.
	silent	Model training progression	1	In scenarios with extensive data and sluggish algorithmic speed, this parameter serves as a tool for monitoring the training progress.
	subsample	A random sampling place with a return sample size	1	Controls the sample size during sampling.1 is the default value, signifying the extraction of 100% of the data at once, whereas a value of 0.1 implies the 10% extraction of the data at a time.
	eta	Iterative decision tree in the learning process	0.1	The step size of the iterative decision tree. Its significance lies in ensuring that each new tree makes an optimal contribution to the overall prediction effectiveness.
	booster	Selection of weak evaluator	Gbtree	During the tree-building process, some trees are discarded, offering a superior overfitting function compared to gradient-boosting trees.
	alpha	Parameters of regular terms	10	With larger values for alpha and lambda, imposing a heavier penalty, the proportion of regularization terms increases, resulting in a lower complexity for the model.
	gamma	Model complexity	2	Critical parameters for mitigating overfitting.

#### 3.7.2. XGBoost parameter configurations.

The evaluation of XGBoost is predominantly influenced by several key factors. These include the iterative decision tree process, the number of decision trees, the choice of a weak evaluator, the XGBoost objective function, the progress of model training, the control of model complexity, parameters of regular terms, and the sample size of random sampling with replacement. Table 4 lists all the XGBoost parameter configurations.

**Table 4:** Parameter settings of XGBoost.

Model	Parameter	Description	Values	Explanation
Attention-customized BiLSTM	Unit	The number of units	99	This parameter is crucial in determining the model’s accuracy and needs to be optimized to reach its ideal value.
	time_step	Time step	99	Assessing the relationship between each input datum and the preceding sequential input data to determine their interdependence.
	num_layers	Number of layers in the unit.	2	The default configuration uses a single layer. If set to two layers, the second layer processes the output from the first layer.
	batch_size	Width of the hidden layer.	64	The amount of data entered concurrently is determined and the model can determine whether the input data is from the same batch.
	Epochs	Number of iterations	64	The computer’s processing power determines the ideal number of iterations.
	Patience	Early Stopping	val_loss 10	To prevent overfitting when the performance on a validation dataset ceases to improve.
	DropOut	Regularization	0.2	To avoid overfitting in neural networks.

## 4. Results

**Fig 12:**
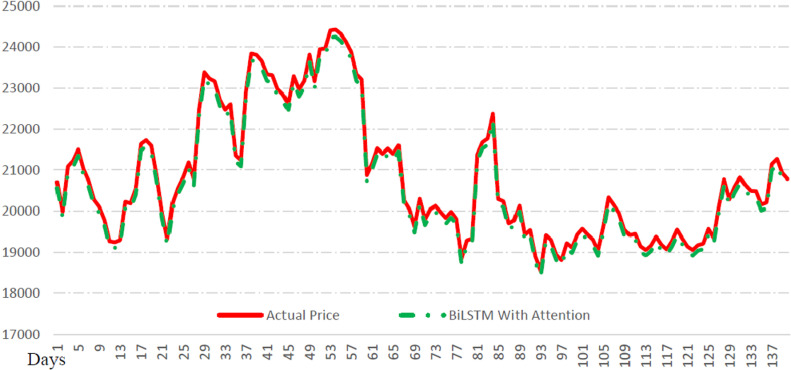
Bitcoin price prediction using BiLSTM with new attention.

**Fig 13:**
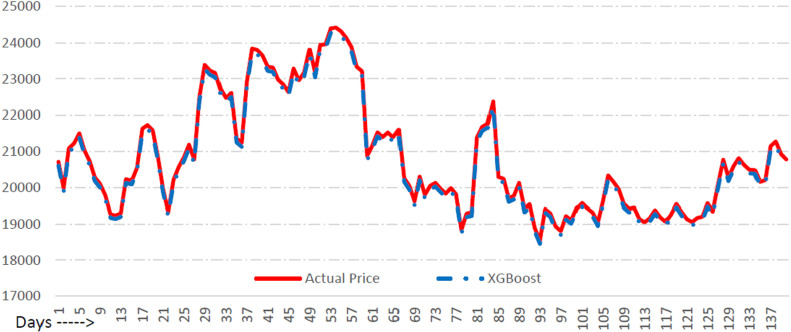
Bitcoin price prediction using XGBoost.

A series of experiments is conducted to check the performance of the proposed ACB-XDE framework by employing two datasets, namely Dataset 1 covering the period between the 1st of October 2014 to the 7th of November 2022, and Dataset 2 extending from 8th November 2022 to the 1st of August 2023. The two datasets consist of the following features Bitcoin, daily opening price, daily closing price, daily trading volume, and daily high and low price ranges. Initially, the attention-customized BiLSTM and XGBoost models are individually trained and tested using Dataset 1, revealing the prediction errors for each model. In Figs 12 and 13, each model’s prediction ability is presented and evaluated based on some key error measures which include Mean Absolute Percentage Error (MAPE), Mean Absolute Error (MAE), and Root Mean Squared Error (RMSE). After computing errors, weights are assigned to the two prediction models using the error reciprocal method. A higher weight is assigned to the model demonstrating a smaller error. Subsequently, these weights are combined using a weighted averaging approach. The primary evaluation index MAPE is utilized in this process, resulting in weights of 0.4252 for the attention-customized BiLSTM and 0.5748 for XGBoost. Consequently, the ensemble of the attention-customized BiLSTM and XGBoost prediction models is formed, and the ensemble ACB-XDE framework undergoes training and testing utilizing linear regression. All the important error metrics show improvement using the ACB-XDE framework. MAPE improved by 27.45% from 0.51 to 0.37. MAE improved by 53.32% from 137.72 to 84.40. Similarly, RMSE improved by 38.59% from 144.73 to 106.14, as shown in Table 5.

**Table 5:** Error Analysis of the proposed framework and evaluation with the state-of-the-art model.

Model	MAPE%	MAE	RMSE
LSTM	1.122	251.52	326.37
Attention-LSTM	1.0	217.25	287.95
XGBoost	0.87	200.09	265.05
BiLSTM	0.86	192.48	248.36
**Ablation study**			
Attention-BiLSTM	0.70	154.23	205.47
Attention-customized BiLSTM	0.51	137.72	144.73
**The proposed ACB-XDE**	**0.37**	**84.40**	**106.14**

**Fig 14:**
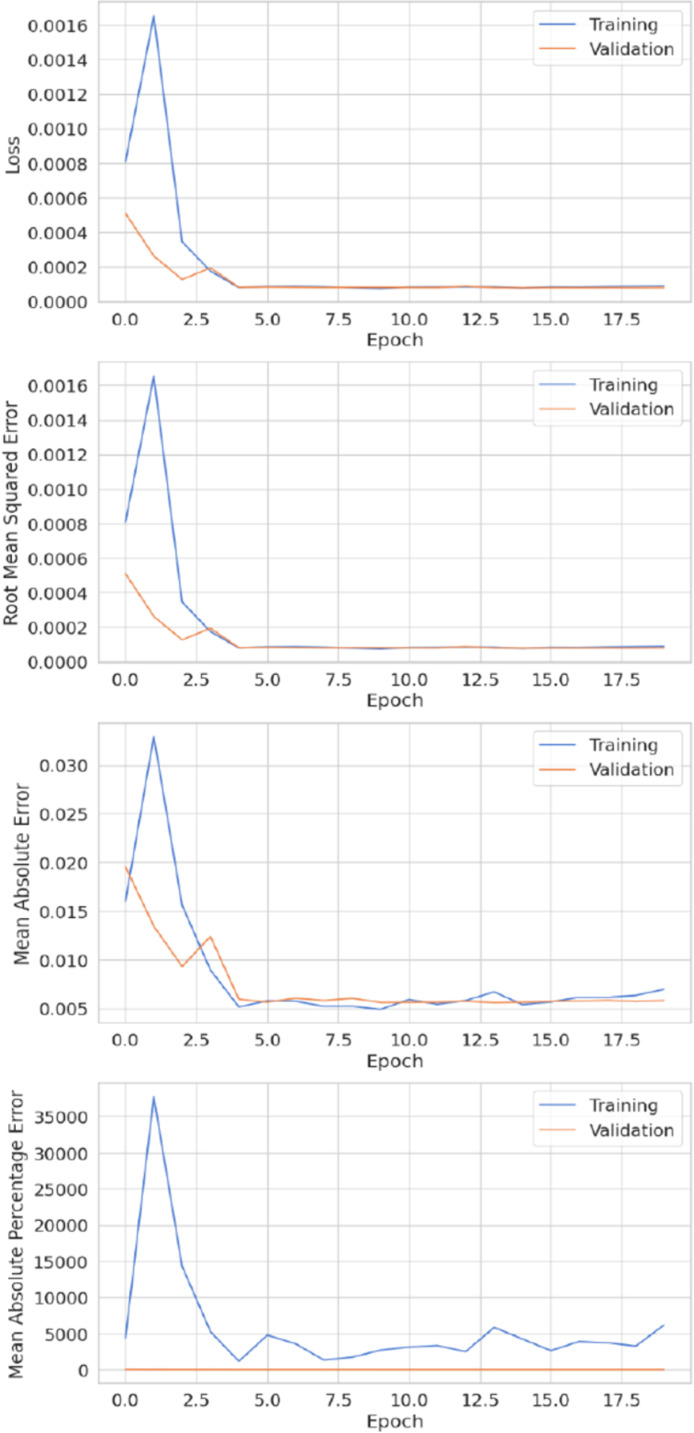
The proposed ACB-XDE framework training and performance evaluation.

Fig 14 illustrates the model’s performance in terms of metrics such as MSE, MAE, MAPE, and RMSE during both the training and validation phases, providing a comprehensive overview of the ensemble framework’s effectiveness. Furthermore, statistical analysis with a CI indicates that the ACB-XDE produces better results, showing a narrower CI range of MAPE (84.4 ± 5.24%).

**Fig 15:**
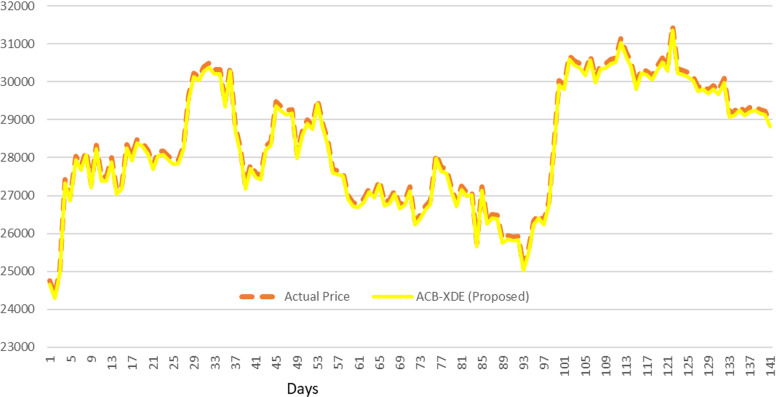
Training and Validating the proposed ACB-XDE framework.

**Fig 16:**
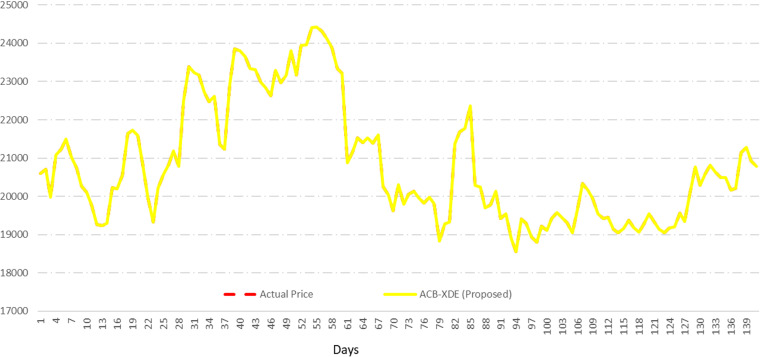
Testing the proposed ACB-XDE framework.

The test results, outlined in Fig 15, further demonstrate the efficacy of the proposed ACB-XDE framework in real-world predictive scenarios. The second dataset (Dataset 2) is used to test the proposed ACB-XDE framework. It is fed to the pre-trained BiLSTM and XGBoost models, and their predictions are subsequently fed into the pre-trained ensemble model of linear regression to obtain the final prediction, as shown in Fig 16.

## 5 Discussion

The customized BiLSTM is employed to address the constraints of BTC-USD data, such as complex dependencies, high volatility, and irregular updates. The customized BiLSTM is chosen for its adeptness at handling long-term dependencies and sequential learning inherent in time series data. However, the utilization of a customized BiLSTM model introduces some challenges like susceptibility to overfitting, especially in the presence of noisy and limited financial data. The computational intensity and resources required for training BiLSTM add a layer of complexity. Moreover, the interpretability of BiLSTM is compromised due to its inherently black-box nature. In response to these limitations, new attention mechanisms and ensemble learning are introduced to improve interpretability by allowing selective focus on critical elements like daily closing price and daily trading volume within the input sequence. While XGBoost is seamlessly integrated into the proposed ACB-XDE framework to address the overfitting. XGBoost improves the ACB-XDE framework by incorporating effective regularization techniques and adapting to non-stationary data. XGBoost brings a complementary strength to the ensemble. The combination of new attention-customized BiLSTM and XGBoost is then consolidated through an ensemble approach. This strategic integration capitalizes on the distinct architecture of both models, fostering a more resilient and accurate prediction model.

**Fig 17:**
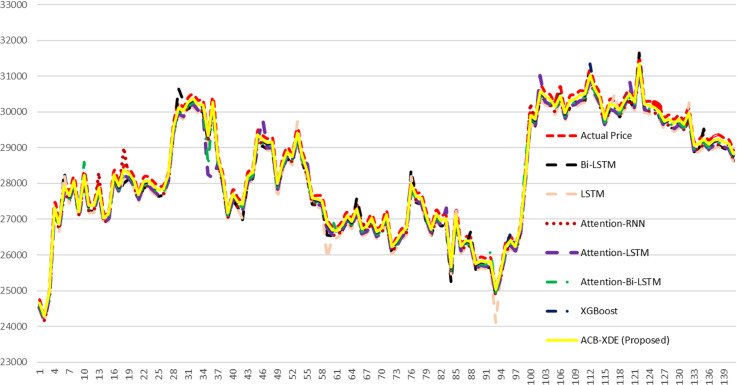
Comparison of the proposed ACB-XDE framework with state-of-the-art models.

**Fig 18:**
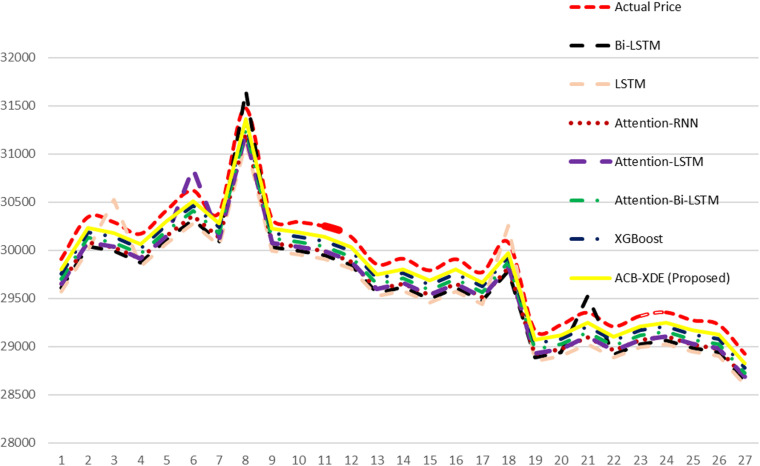
The sub-portion of the above Fig 17.

**Fig 19:**
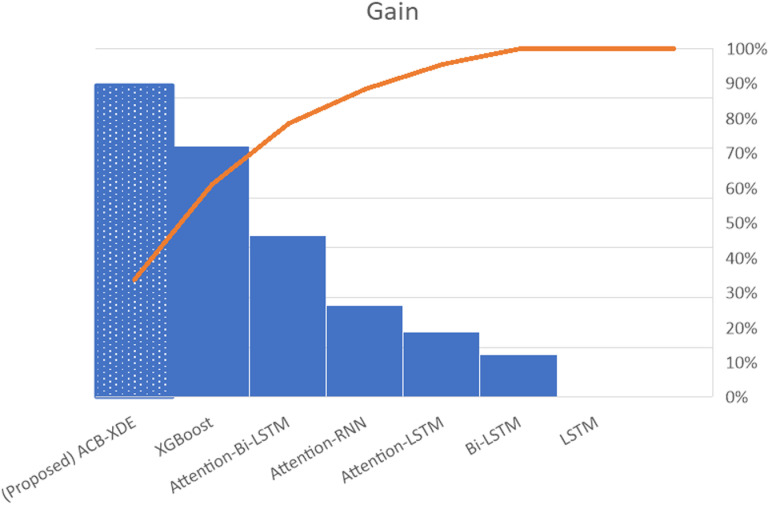
The proposed ACB-XDE framework and state-of-the-art models gain.

**Fig 20:**
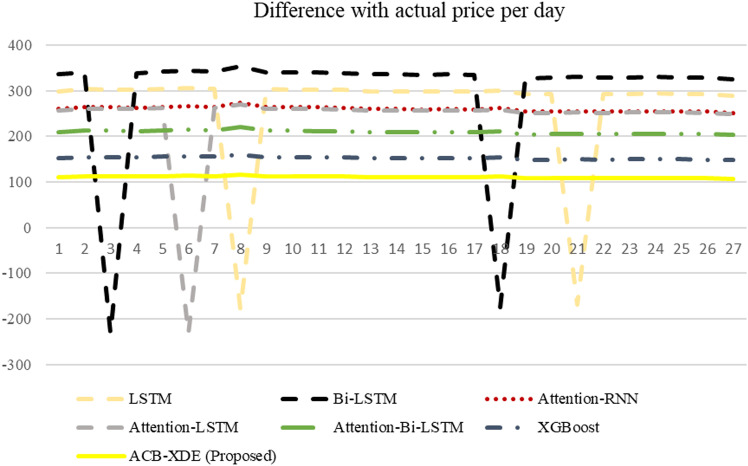
Difference of the proposed framework and state-of-the-art models with actual price per day.

The efficacy of the proposed ACB-XDE framework is validated by employing LSTM, BiLSTM, attention-LSTM, attention-BiLSTM, attention-customized BiLSTM, XGBoost, and the proposed ACB-XDE framework for data prediction. Table 5 presents the Error Analysis of the proposed framework alongside state-of-the-art models, as shown in Fig 17. Additionally, Fig 17 is locally enlarged in Fig 18 to provide a clearer observation of the trend and proximity between each model’s forecast results and the actual values. Similarly, Table 6 offers a comparative analysis of previous studies on Bitcoin across various distributions. This comparative analysis serves to underscore the performance of the proposed ACB-XDE framework in generating predictions. Additionally, the detailed performance gain and difference in price per day of the proposed ACB-XDE framework over the existing state-of-the-art models are shown in Figs 19 and 20, respectively. Moreover, Table 7 presents the 95% CI for the MAPE of the state-of-art the models including the proposed framework. The proposed ACB-XDE framework has the smallest interval range MAPE (84.4 ± 5.24%), which indicates that the predictions made by the proposed ACB-XDE framework are better than those predicted by LSTM, Attention-LSTM, XGBOOST, and others.

**Table 6:** Error analysis of the previous studies employed on bitcoin at various distributions.

Model	MAPE%	MAE	RMSE
LSTM vs. ARIMA [43]	–	–	197.515
ARIMA 1 Day [44]	0.87	–	–
MICDL [45]	–	170	265.05
CNNs + LSTM [46]	2.3	209.89	258.31
LSTM 1 Day		875.06	1203.97
LSTM 3 Day		926.96	1311.71
LSTM 7 Day		1191.05	1645.36
CNN 1 Day		801.5	1107.77
CNN 3 Day		1363.85	1699.56
CNN 7 Day [47]		1197.38	1670.93

**Table 7:** MAE confidence interval variation.

Model	MAPE
LSTM	1.122 ± 12.86%
Attention-LSTM	1 ± 11.65%
XGBoost	0.87 ± 9.97%
BiLSTM	0.86 ± 10.84%
Attention-BiLSTM	0.7 ± 8.56%
Attention-Customized BiLSTM	0.51 ± 7.38%
The Proposed ACB-XDE	0.37 ± 5.24%

### 5.1. Observations and trends from results

#### 5.1.1. Trend comparison: Predicted values compared to actual values.

a) Fig 17 presents a visual comparison between real values and predictions derived from various models, including LSTM, attention-LSTM, BiLSTM, attention-BiLSTM, New attention-BiLSTM, XGBoost models, and ACB-XDE. In contrast to the BiLSTM model, which achieves lower accuracy, the proposed ACB-XDE framework achieves the highest accuracy.
b) The ACB-XDE’s curve closely aligns with the real value curve, showcasing superior fitting effects and a consistent trend.
c) The ACB-XDE performs well in terms of accuracy and sensitivity to changes in proportionality. Fig 17 is locally enlarged in Fig 18 to provide a better understanding of patterns and the proximity between predicted and actual values.

#### 5.1.2 . Localized enlargement (6 July - 01 August 2023).

Fig 18 highlights the enhanced consistency of the proposed ACB-XDE framework’s curve with the real value curve, surpassing benchmarks and other models. Figs 17 and 18 respectively demonstrate the better prediction accuracy of the proposed ACB-XDE framework compared to other models under consideration.

### 5.2. Comparison and analysis of errors

Table 5 outlines MAPE, MAE, and RMSE values for the six models. This analysis unveils valuable insights into their comparative performance.

a) Model selection impact: The BiLSTM model surpasses LSTM, showcasing lower errors, and emphasizing its pivotal role in this study.
b) Attention mechanism influence: A thorough model comparison highlights the profound influence of the attention mechanism on prediction accuracy across LSTM, attention-LSTM, BiLSTM, attention-BiLSTM, and attention-customized BiLSTM models.
c) Benchmark model performance: XGBoost surpasses LSTM and BiLSTM, demonstrating superior prediction with the smallest error. Integrating XGBoost with attention-BiLSTM significantly enhances accuracy.
d) Combined models superiority: Table 5 data highlights the ACB-XDE’s supremacy, presenting minimal MAPE, MAE, and RMSE values at 0.37, 84.40, and 106.14, respectively. Moreover, it has a narrower CI MAPE range (84.4 ± 5.24%). This highlights the ensemble approach’s effectiveness in minimizing overall prediction errors, surpassing individual models for better accuracy.

## 6. Conclusion

This paper introduces a novel ACB-XDE framework aimed at predicting the daily price of Bitcoin. Bitcoin is very complex and volatile. Bitcoin is chosen for this study because of its high volatility and significant impact on financial markets. Bitcoin price prediction provides a robust test case for the proposed ACB-XDE framework. The proposed ACB-XDE framework combines an attention-customized BiLSTM, which incorporates a new attention block, with a modified XGBoost algorithm. These models leverage the learning capabilities of complex sequential dependencies and recognize trends in speculative market behavior. The attention mechanism in the customized BiLSTM assigns weights dynamically and focuses on important features, with a particular emphasis on daily closing prices and daily trading volumes. XGBoost helps prevent overfitting and makes the algorithm more efficient, which improves overall predictive accuracy. In this research, the performance of the proposed ACB-XDE framework is tested against several state-of-the-art models, including new attention-BiLSTM, XGBoost, LSTM, BiLSTM, attention-LSTM, and attention-BiLSTM. The ACB-XDE framework shows improved performance in handling complex sequential dependencies, high volatility, and dynamic patterns. Empirical validation on the BTC-USD dataset shows ACB-XDE’s MAPE of 0.37%, MAE of 84.40, and RMSE of 106.14, outperforming the existing models. Compared to the new attention-BiLSTM, the ACB-XDE framework significantly reduced error metrics: MAPE decreased from 0.51 to 0.37 (around 27.45% reduction), MAE from 137.72 to 84.40 (about 53.32% reduction), and RMSE from 144.73 to 106.14 (about 38.59% reduction). Statistical analysis, such as CI confirms the improved performance of ACB-XDE. It has the smallest CI range (84.4 ± 5.24%). These improvements highlight the improved predictive accuracy of the ACB-XDE framework. In summary, the ACB-XDE framework effectively addresses the limitations of traditional and modern forecasting methods, showing better generalization capabilities. Future research directions include evaluating the framework across diverse datasets, expanding the array of evaluation indicators, and optimizing parameters and hyperparameters using methodologies like Bayesian optimization. Additionally, incorporating external influences, legal and regulatory aspects, and seasonality trends as input features will further refine the model. These strategic considerations aim to extend the application of the ACB-XDE framework to various fields, thereby broadening its impact and contributing to advancements in predictive modeling across multiple domains. Moreover, the proposed framework will be employed in multi-step-ahead forecasting.
